# Temporal Trends of Urinary Phthalate Concentrations in Two Populations: Effects of REACH Authorization after Five Years

**DOI:** 10.3390/ijerph15091950

**Published:** 2018-09-06

**Authors:** Giovanna Tranfo, Lidia Caporossi, Daniela Pigini, Silvia Capanna, Bruno Papaleo, Enrico Paci

**Affiliations:** Department of Occupational and Environmental Medicine, Epidemiology and Hygiene, INAIL-National Institute for Insurance against Accidents at Work, 00078 Monteporzio Catone, Italy; g.tranfo@inail.it (G.T.); d.pigini@inail.it (D.P.); s.capanna@inail.it (S.C.); b.papaleo@inail.it (B.P.); e.paci@inail.it (E.P.)

**Keywords:** REACH regulation, phthalates, biomonitoring

## Abstract

Phthalates are widely used in the industrial manufacture of many products. Some phthalates have shown reproductive toxicity in humans, acting as endocrine disruptors, so they were included in the authorization process defined in Reg. CE 1907/2006 (REACH). Two groups of population were recruited, before and after the inclusion of some phthalates in the authorization list in REACH: the first group of 157 volunteers was studied in 2011 and the second, 171 volunteers, in 2016. Each subject completed a questionnaire about personal lifestyle, working activities and use of chemical products. The main urinary metabolites of five phthalates were analyzed by HPLC/MS/MS: mono(2-ethylhexyl)phthalate (MEHP) and mono(2-ethyl-5-hydroxyhexyl) phthalate (MEHHP) for di(2-ethylhexyl)phthalate (DEHP) exposure; monoethylphthalate (MEP) for diethylphtahate (DEP); monobenzylphthalate (MBzP) for butylbenzylphtahalate (BBP) and dibenzylphthalate (DBzP), mono-*n*-butylphthalate (MnBP) for butylbenzylphtahalate (BBP) and di-*n*-butylphthalate (DnBP). The results show a significant difference for all metabolites between the two periods, with the exception of MEP in women. The comparison of the two sets of results shows a decrease in urinary metabolites excretion from 2011 to 2016, statistically significant for the three phthalates included in Annex XIV of REACH. DEP, not currently included in the list for authorization, maintains a constant presence in the daily life of the population, particularly for women.

## 1. Introduction

The diesters of phthalic acid are a group of similar molecules, widely used since the 1930s in many commercial industrial product manufacturing processes, the most important of which is the production of plastics. The inclusion of phthalates in rigid polymers (such as PVC) allows the production of more flexible plastics. They are also used in construction materials, vinyl flooring, food packaging and medical devices. This widespread use results in a possible human exposure to phthalates [1]. 

Phthalates having a smaller molecular structure and lower molecular weight are more soluble in water and are therefore used as industrial solvents, in the composition of cosmetics and pharmaceuticals, or as insecticides; this is the case, for example, of diethyl phthalate (DEP) and di-*n*-butyl phthalate (DnBP) [2,3]. In particular, DEP is the phthalate of choice used in the cosmetic and personal hygiene products industry, both for adults and for children/infants [4], while DnBP is usually used as excipient in pharmaceuticals products [5,6], and it was present in cosmetics, particularly nail care products for adults [7]; in Europe this last use is forbidden by the EU cosmetics directive, Dir. 76/768/EEC, where other forbidden phthalates were also listed: di(2-ethylhexyl)-phthalate (DEHP), benzylbutylphthalate (BBP), bis2-methoxyethylphthalate, *n*-pentylisopentyl-phthalate, di-*n*-pentylphthalate, diisopentylphthalate and di (*n*-butyl) phthalate (DnBP). Historically, DEHP is the phthalate produced in the largest quantities around the world, about 2 million tons per year [8]. Nevertheless, DEHP has been recently replaced in numerous production cycles by diiso-nonylphthalate (DiNP), including the production of flexible PVC, because of its dangerous characteristics and in particular its toxicity for human reproduction [9]. In the scientific literature, some authors have shown that also infants are exposed to some phthalates, possibly through breast milk, infant formulae and baby food [10,11,12]. Furthermore the possibility of phthalate migration from packaging to food or beverages represent a possible risk of contamination [13,14] and therefore of human exposure.

Some phthalates act as endocrine disruptors, particularly interacting with estrogen receptors and interfering on hormone homeostasis. In particular, DnBP and DEHP have shown to cause antiandrogenic effects in in vivo experiment [15], with decreased testosterone concentrations and sperm production [16,17]. 

In addition to the toxic effects for the endocrine system, effects on kidney and liver have been highlighted [17,18], as well as the possibility that exposure to high concentrations of phthalates may affect the etiology of certain tumors [19], associated with higher fetal death or childhood malformations [17,20].

The exposure of the general population to these substances was assessed using biomonitoring studies both in the United States and in Europe [21,22,23]. Since 1979, in Europe, the legislation started to protect the more vulnerable consumers from this type of exposure by means of the European directive 79/769/CEE, in which the maximum permitted concentrations of six phthalates in toys and children products were stated; these indications currently remain as restrictions. 

Presently, the European Regulation CE 1907/2006, named Registration, Evaluation and Authorization of Chemicals (REACH), that contains these restrictions, also includes some phthalates in a list of “substances of high concern”. We report in Table 1 the list of phthalates that are on the candidate list for authorization and/or in the Annex XIV of REACH, with details about the date of inclusion and the reason for it. This action will gradually bring to the substitution of these chemicals in the involved industrial processes, with alternative substances, even if it’s not simple to find chemicals with the same industrial characteristics and lower or no health effects. At the moment in fact there’s a heated scientific debate in Europe about the safety of replacement chemicals.

Phthalates are not persistent molecules from an environmental point of view, as they tend to degrade easily; at a biochemical level, similarly, they rapidly produce the respective monoester metabolites, which follow a mainly urinary excretory pathway. High molecular weight molecules trend to further react giving oxidative products [24], and/or partially undergo glucuronidation reactions and are excreted mainly in the urine, and, to a lesser extent, in the feces [25]. The environmental contamination levels that are reported in the scientific literature are justified by a continuous production of the phthalates and wide dispersion, resulting in their presence in dust, soil, indoor and outdoor air.

In order to evaluate the internal dose of phthalates, biomonitoring is the preferred strategy as it offers the advantages of integrating exposure through all routes (e.g., inhalation, dermal, ingestion). Furthermore the urinary metabolites analysis permit to carry out a more precise measurement with respect to the parent compounds and, even more important, the risk of accidental contamination of samples during collection, storage and analysis (due to the use of plastic devices and to the environmental contamination) is greatly reduced [26,27]. Therefore, the urinary metabolites became the most used biological indicators of exposure, both for general population and for particular group of subjects [28,29,30,31]. The specific metabolic route of each phthalate, and the corresponding monoester metabolite, is related to the size of the aryl and/or alkyl chains present in the molecular structure.

The aim of the present study was to evaluate the exposure due to lifestyle to five phthalates, namely DEHP, DEP, DnBP, BBP and dibenzylphthalate (DBzP), by means of the analysis of the respective metabolites in urine samples collected with a five year gap (2011–2016) in two population groups. We compared the two series of biomonitoring data, gathered before and after the phthalates were inserted into annex XIV of REACH, in order to understand if the application of the authorization process could bring to a real reduction of the general population exposure.

For the present study DEHP, DEP, DnBP, BBP, DBzP were selected, due to their human toxicity but also to their wide presence in living environments. DEHP, DnBP and BBP are included in Annex XIV, but not DEP and DBzP. We considered DEP because it is largely used in cosmetics and cleaning and care products, and DBzP, that is an ingredient of drug capsules, as its metabolite, MBzP, is also the metabolite of BBP. We report in Table 2 the list of all the phthalates considered in the present study, with details about their main urinary metabolites and their presence in processes and products, as well as possible exposure sources [32].

## 2. Materials and Methods 

We ensured the respect for human dignity, protected the value, rights and interests of the participants to the research project according to Declaration of Helsinki and following the European and Italian Law about ethical principles and data protection/management and privacy. 

For these purposes, all the enrolled subjects were volunteers, of both sexes, adult workers of different tasks in several workplaces, not belonging to vulnerable populations, not minors, without diagnosed pathologies, able to perform their working task according to their occupational physician. All subjects gave their informed consent to participate to the study. They were tested in an occupational medicine clinic, during the periodic health surveillance for the prevention of risks for safety and health at work. They represent a general population sample, from professionals graduated to blue-collar workers. The sampling strategy included a preliminary phase of illustration of the project, with adhesion through the signing of the informed consent by the subjects meeting the inclusion criteria, then the definition of the medical examination time and contextual collection of the spot urine samples.

We enrolled the first sample of population in 2011, consisting in 157 volunteers (83 women and 74 men) [39] and the second sample in 2016, 171 volunteers (111 women and 60 men). All of them were living in Central Italy. Each subject was asked to complete a questionnaire for collecting information about personal lifestyle, food habits, smoking, drug use, working activities, hobbies and use of chemical products.

In order to identify possible sources of exposure we examined also the following variables: the habit to store fat foods in plastic containers, because is documented that a migration between plastic containers and food is greater and faster in case of fat food [40], and the habit of eating fish, especially if rich in fat like mackerel, salmon and eel, and a regular intake of canned foods [41]. 

The inclusion criteria were:

1. Healthy volunteers not occupationally exposed to phthalates;
2. Subjects having creatinine concentrations between 0.3 and 3 g/L [42] because when the urine is too diluted or concentrated this could affect the accuracy of the analytical results [43,44].

Urine samples were collected in sterile polypropylene containers, and, after being transported in our laboratory at room temperature, quickly frozen, and stored at −20 °C until analysis. The analysis was carried out using the procedure previously published [31], using HPLC/MS/MS in order to determine the urinary concentrations of the following metabolites: mono(2-ethylhexyl)phthalate (MEHP), mono(2-ethyl-5-hydroxyhexyl)phthalate (MEHHP), monoethylphthalate (MEP), mono-benzylphthalate (MBzP), and mono-*n*-butylphthalate (MnBP). We summed the molar concentrations of the two DEHP metabolites to calculate the concentration related to DEHP exposure.

Statistical analysis was performed with the software SPSS^®^ 19.0 (IBM^®^ Corporation, New York, NY, USA). To study the distribution of the data the Shapiro–Wilk test was used, with a significance of 0.05. The application of the Shapiro- Wilk test indicated that the urinary phthalates’ value were not normally distributed (*p* < 0.05), and therefore non-parametric tests were applied for the evaluation of the difference between the two periods. The urinary concentrations of phthalates were analyzed to determine possible differences between the two groups of data using the Mann–Whitney, nonparametric test, with a significance of 0.05.

## 3. Results

In Table 3 the population characteristics and the information collected with the questionnaire are reported separately for the two biomonitoring campaigns.

In Table 4 the Limit of Detection for each metabolite and the respective detection frequency over the LOD for all samples (both campaigns) are reported.

**Figure 1:**
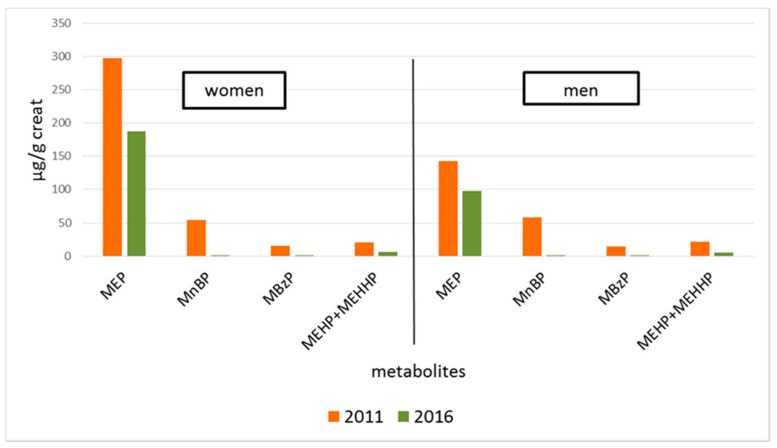
Comparison between periods, median data of urinary concentrations of phthalates’ metabolites in women and in men (MEP: monoethylphthalate; MnBP: mono-*n*-butylphthalate; MBzP: monobenzylphthalate; MEHP: mono(2-ethylhexyl)phthalate; MEHHP: mono(2-ethyl-5-hydroxyhexyl)phthalate).

The comparison between the two studies is graphically reported in Figure 1, stratified by gender, in terms of median values.

The analytical results of the urinary phthalate metabolites for all the volunteers and for the two campaigns are reported in Table 5 and Table 6. The results presented in Table 5 and Table 6 show a statistically significant difference (*p* < 0.05) for all metabolites, both for men (median value, in µg/g creatinine, of: MEP 49.9 vs. 21.6; MnBP 37.6 vs. 0.0; MBzP 4.8 vs. 0.0; ∑DEHP metabolites 14.1 vs. 3.1) and for women (median value, in µg/g creatinine, of: MnBP 38.8 vs. 0.0; MBzP 7.0 vs. 0.5; ∑DEHP metabolites 15.6 vs. 4.5) between the two periods, with the exception of MEP in women (median value 73.1 vs. 49.9 µg/g creatinine); the group whose samples were taken in the more recent period presents an overall lower urinary metabolites concentration of phthalates than the other group.

With regard to the dosage of the MEP, it should be remembered that the DEP is one of the phthalates, among those taken into consideration, which is not subject to the REACH authorization process. This means that DEP is still widely used in everyday products, many of which are cosmetics and personal hygiene products; this probably justifies a higher concentration of the MEP in the urine of women and a not statistically significant reduction in the five years’ time lapse.

A comparison study between the two genders was also conducted, within the same period of recruitment and no significant differences emerged in 2011, while in 2016 MEP and MBzP were significantly (Mann-Whitney, *p* < 0.05) higher in women than in men (median value, in µg/g creatinine of: MEP 49.9 vs. 21.6; MBzP 0.5 vs. 0.0).

Besides, some considerations about the possible sources of exposure were done using the data from the questionnaires. The application of a statistical test to smoking and non-smoking subjects (all data) did not show any significant difference (Kruskall–Wallis > 0.05). The same test, performed between subjects consuming canned food at least weekly and all the others, showed higher concentrations of MEHHP (median value, in µg/g creatinine: 16.0 vs. 3.2) in people consuming canned food, in the first group of subjects (enrolled in 2011); the molar sum of MEHHP and MEHP urinary concentrations (as they are both metabolites of the same phthalate) are, on average, higher in people using plastic containers to store fatty foods, in both groups (median value, in µg/g creatinine, in 2011: 18.7 vs. 4.6; in 2016 3.9 vs. 0.2).

## 4. Discussion

Our results suggest that non-occupational exposures to phthalates of the studied subjects, without doubt, occurred in the considered geographical area; the exposure concentrations are quite similar to those measured in different countries for some metabolites. We report in Table 7 and Table 8 a comparison of urinary concentrations of phthalate metabolites found in different populations, stratified for gender. 

More numerous studies on women than on men can be found in the literature, and papers that focus on both sexes sometimes showed results without a difference between genders [45]; this can be a problem for the interpretation of data, particularly because gender is an important variable for exposure.

Looking for scientific articles reporting temporal trends in phthalate exposure we can found a decreased concentration, in the last decade, both in USA [46,47] and in Europe [48,49], with values decreasing from 17% to 42%; the reason for it was indicated in the legislative activity in western countries. 

In particular it was observed that exposure to the “alternative chemicals” was increasing while the exposure to the original compounds was lowered [45,47,49,50]. The questionnaire allowed us to highlight some of the possible common sources of exposure to phthalates in everyday life, particularly the use of plastic containers for the storage of fat foods and eating canned foods [41].

### 4.1. Strengths and Limitations of the Study

The strength of the present study is certainly to have proposed a biomonitoring survey of exposure to phthalates in Italy, for a general population group. Currently, phthalate dosing studies, in Italy, are aimed to investigate specific correlations with pathologies [55,56], selecting samples of specific subjects, mainly children [55,56,57,58] and pregnant [59] or menopausal [60] women, and not to a more general exposure characterization. A second strength is to produce data, useful for the scientific debate, to confirm the temporal trends of phthalate in Europe, after the REACH application.

Considering the rate of metabolism and excretion of phthalates, the individual concentration can show daily and/or weekly variability, which a sampling of spot urine cannot evaluate. Testing a single spot urine sample may therefore represent a limitation of the study. Other limitations are showing results only from a population of working adults, with a range of age 28–50, and enrolling subjects living in a specific geographic area, that make the generalization of results to all the population, also in considerations of possible regional variability, largely approximate. Concluding, it is certainly desirable to increase the number of subjects recruited in order to be able to extend the data and increase their significance.

## 5. Conclusions

The comparison of the two sets of results clearly shows the dramatic decrease in urinary metabolites excretion from 2011 to 2016, for the three phthalates included in Annex XIV of REACH.

The analytical method performances render it suitable for the quantitative determination of the analytes in urine samples of the general population. 

DEP, not currently included in the candidate list for authorization, maintains a constant presence in the daily life of the population; this is more important for the female gender, with high probability in consideration of the larger use of cosmetics and products for personal hygiene.

It is important to underline how DEP is included in the European classification of endocrine disruptors in category 1, therefore as a “substance of high concern” with regards of possible endocrine interference for humans. This suggests that this phthalate will certainly require to be further studied and reconsidered by the legislators to assess the possible risk for human health, particularly for women, and consequently it will be needed to reduce the exposure sources in the near future.

Future research should be aimed to increase the number of enrolled subjects for a stronger statistical significance of the data and to allow the identification of the possible exposure sources for the general population.

We think that the downtrend in the urinary excretion of phthalate metabolites is clearly a consequence of their less widespread use and of the lower environmental contamination of the last few years, reflecting the application of the authorization process to some phthalates starting from 2013, and confirming the efficacy of REACH legislation.

## Figures and Tables

**Table 1:** Phthalates of “high concern” and information about the authorization due for Reg. CE 1907/2006.

Substance	Acronymous	In List for Authorization from	In Annex XIV of REACH from	Cause for Being in the List
Di-(2-ethylhexyl)-phthalate	DEHP	28 October 2008	21 August 2013	Endocrine disrupters Toxic for reproduction
Di-(*n*-butyl) phthalate	DnBP	28 October 2008	21 August 2013	Endocrine disrupters Toxic for reproduction
Benzylbutyl phthalate	BBP	28 October 2008	21 August 2013	Endocrine disrupters Toxic for reproduction
Diisobutyl phthatale	DiBP	13 October 2010	21 August 2013	Endocrine disrupters Toxic for reproduction
Diisopentyl phthalate	DIPP	19 December 2012	04 January 2019	Toxic for reproduction
Dipentyl phthalate	DPP	19 December 2012	04 January 2019	Toxic for reproduction
*n*-Pentylisopentyl phthalate	nPiPP	19 December 2012	04 January 2019	Toxic for reproduction
Bis(2-Methoxyethyl) phthalate	DMEP	19 December 2011	-	Toxic for reproduction

**Table 2:** List of the phthalates considered in the present study with details on their main metabolites and use.

Substance	Acronymous	Main Metabolites ^1^	Where You Can Find It
Di-(2-ethylhexyl)-phthalate	DEHP	MEHP (6%) MEHHP (33–49%) 5-oxo-MEHP (14%) [22,33,34]	Production of PVC ^2^ and vinyl chloride resins, where it is added to plastics to make them flexible. Adhesives and sealants, arts, crafts and hobby materials. Building/construction materials, electrical and electronic products, fabric, textile and leather products, paints and coatings, plastic and rubber products, playground and sporting equipment. It’s a possible food contaminant for indirect contact.
Di-(*n*-butyl)phthalate	DnBP	MnBP (84%) [35]	Production of plastics to help make it soft and flexible. Shower curtains, raincoats, food wraps, bowls, car interiors, vinyl fabrics, floor tiles, and other products. Adhesives and sealants, explosive materials, floor coverings, ink, toner and colorant products. Plastic and rubber products, excipient in drugs.
Benzylbutyl phthalate	BBP	MnBP (44%) MBzP (16%) [36]	Adhesive and sealants, floor coverings
Dibenzyl phthalate	DBzP	MBzP (%not defined) [37]	Ingredient in drugs for: disorders of the urinary system, prostate, bladder; dermatological disorders; skeletal disorders; antipsoriatics; arthritis, arthrosis, antiasthmatics, muscular and neuromuscular system disorders, nervous system disorders and many other type of drugs.
Diethyl-phthalate	DEP	MEP (70%) [38]	Odor agents, plasticizers, adhesives and sealants, air care products, automotive care products, cleaning and furnishing care products, ink, toner and colorant products, laundry and dishwashing products, paints and coatings, personal care products, plastics and rubber products

^1^ MEHP: mono(2-ethylhexyl)phthalate, MEHHP: mono(2-ethyl-5-hydroxyhexyl)phthalate, MEP: monoethylphthalate, MBzP: monobenzylphthalate, MnBP: mono-*n*-butylphthalate, 5-oxo-MEHP: 5-oxo mono(2-ethylhexyl)phthalate; ^2^ PVC: polyvinyl chloride.

**Table 3:** Questionnaire results.

Characteristics	2011		2016	
	Women (*n* = 83)	Men (*n* = 74)	Women (*n* = 111)	Men (*n* = 60)
Age (SD ^1^)	36.5 (7.2)	40.4 (7.3)	42.4 (8.3)	41 (9.2)
Smoking (% current)	15.7	32.4	39.3	47.5
Regular and occasional alcohol intake (%)	37.3	58.1	58	90.2
Area of residence (%)				
Urban	73.5	71.6	63.4	57.4
Rural	21.7	20.3	36.8	26.2
Coast	4.8	8.1	6.3	16.4
Other	-	-	3.5	-
Use of plastic containers for fat food storage (%)				
Never	59.0	54.1	59.3	55.2
Daily	10.8	9.5	12.3	7.1
weekly	15.7	17.6	15.4	14.2
monthly	14.5	18.8	13.0	23.5
Use of canned foods at least weekly (%)	31.3	31.1	25.9	23.0
Eating fat fish at least weekly (%)	26.5	25.7	58	68.9
Job (%)				
Office/school	35.7	28.6	30.8	23.3
Trade	26.9	32.3	12.6	15.5
Craftsman/manual worker	1.2	9.5	4.3	17.4
Cleaning man/woman	12.0	2.7	26.4	8.2
Hairdresser/beautician	3.6	1.4	7.5	
Armed forces	1.2	12.2		18.9
Healthcare/Laboratory	18.1	13.5	12.5	10.1
Other	0.1	-	5.9	6.6

^1^ SD: standard deviation.

**Table 4:** Limits of Detection (LOD) and detection frequencies.

LODs (µg/L)		MEP ^1^		MnBP ^2^		MBzP ^3^		MEHHP ^4^		MEHP ^5^	
		1.0		3.0		0.2		1.0		1.0	
		2011	2016	2011	2016	2011	2016	2011	2016	2011	2016
Men	N ^6^ < LOD	22	19	1	54	0	33	4	6	0	49
	% ^7^ > LOD	71	68	99	10	100	45	95	90	100	18
Women	N ^6^ < LOD	0	11	0	99	2	38	0	13	35	85
	% ^7^ > LOD	100	90	100	11	98	66	100	88	57	23

^1^ MEHP: mono(2-ethylhexyl)phthalate; ^2^ MnBP: mono-*n*-butylphthalate; ^3^ MBzP: monobenzylphthalate; ^4^ MEHHP: mono(2-ethyl-5-hydroxyhexyl)phthalate; ^5^ MEP: monoethylphthalate; ^6^ N: number of samples <LOD; ^7^ %: detection frequencies.

**Table 5:** Results of urine analysis of women (µg/g creatinine).

	MEP ^4^		MnBP ^5^		MBzP ^6^		MEHP ^7^ + MEHHP ^8^	
	2011 ^1^	2016 ^2^	2011 ^1^	2016 ^2^	2011 ^1^	2016 ^2^	2011 ^1^	2016 ^2^
Average (SD ^3^)	297.7 (881.1)	187.7 (321.0)	54.5 (55.7)	1.3 (5.3)	15.0 (21.9)	1.3 (3.7)	20.9 (17.0)	5.9 (5.6)
Median	73.1	49.9	38.8	0.0	7.0	0.5	15.6	4.5
5th percentile	6.4	0.0	4.8	0.0	0.7	0.0	3.6	0.8
95th percentile	1177.9	781.3	163.0	6.4	72.8	5.1	61.4	16.9

^1^ 83 women in 2011; ^2^ 111 women in 2016; ^3^ SD:standard deviation; ^4^ MEP: monoethylphthalate; ^5^ MnBP: mono-*n*-butylphthalate; ^6^ MBzP: monobenzylphthalate; ^7^ MEHP: mono(2-ethylhexyl)phthalate; ^8^ MEHHP: mono(2-ethyl-5-hydroxyhexyl)phthalate.

**Table 6:** Results of urine analysis of men (µg/g creatinine).

	MEP ^4^		MnBP ^5^		MBzP ^6^		MEHP ^7^ + MEHHP ^8^	
	2011 ^1^	2016 ^2^	2011 ^1^	2016 ^2^	2011 ^1^	2016 ^2^	2011 ^1^	2016 ^2^
Average (SD ^3^)	142.7 (243.6)	97.7 (218.1)	57.7 (62.6)	1.4 (7.2)	14.5 (27.8)	0.9 (2.2)	21.1 (26.0)	5.7 (7.1)
Median	49.9	21.6	37.6	0.0	4.8	0.0	14.1	3.1
5th percentile	4.2	0.0	7.3	0.0	0.8	0.0	3.8	0.0
95th percentile	637.9	406.7	145.6	4.8	51.7	3.0	62.1	20.1

^1^ 74 men in 2011; ^2^ 60 men in 2016; ^3^ SD: standard deviation; ^4^ MEP: monoethylphthalate; ^5^ MnBP: mono-*n*-butylphthalate; ^6^ MBzP: monobenzylphthalate; ^7^ MEHP: mono(2-ethylhexyl)phthalate; ^8^ MEHHP: mono(2-ethyl-5-hydroxyhexyl)phthalate.

**Table 7:** Comparison among biomonitoring median data in different geographical areas—women (µg/g creatinine).

Country	Italy [This Study]	Italy [This Study]	Taiwan [51]	Israel [52]	USA [46]	USA [46]	Sweden [48]	the Netherlands [53]	Germany [54]
**Sampling year**	**2011**	**2016**	**2005–2006**	**2006**	**1999–2000**	**2011–2012**	**2009–2014**	**2004**	**2005**
**N **	**83 **	**111**	**76**	**19**	**1326**	**1229**	**178**	**100**	**27**
**phthalates**									
MEP ^2^	73.1	49.9	68.0	140.5	123.0 ^1^	51.8 ^1^	24.3	112.0 ^1^	-
MnBP ^3^	38.8	0.0	195.0	45.9	28.6 ^1^	9.8 ^1^	42.7	43.2 ^1^	46.8
MBzP ^4^	7.0	0.5	3.7	9.6	11.0 ^1^	5.87 ^1^	8.76	8.9 ^1^	7.6
∑DEHP ^5^ met.	15.6	4.5	60.8	31.3	3.36 ^1^	1.70 ^1^	11.4	21.2 ^1^	4.3

^1^ geometric mean; ^2^ MEP: monoethylphthalate; ^3^ MnBP: mono-*n*-butylphthalate; ^4^ MBzP: monobenzylphthalate; ^5^ DEHP: di(ethylhexyl)phthalate.

**Table 8:** Comparison among biomonitoring median data in different geographical areas—men (µg/g creatinine).

Country	Italy [This Study]	Italy [This Study]	USA [46]	USA [46]	Germany [54]
**Sampling year**	**2011**	**2016**	**1999–2000**	**2011–2012**	**2005**
**N **	**74**	**60**	**1215**	**1258**	**23**
**phthalates**					
MEP ^2^	49.9	21.6	92.8 ^1^	35.8 ^1^	-
MnBP ^3^	37.6	0.0	17.3 ^1^	7.61 ^1^	41.4
MBzP ^4^	4.8	0.0	9.14 ^1^	4.5 ^1^	5.1
∑DEHP ^5^ met.	14.1	3.1	2.89 ^1^	1.41 ^1^	4.3

^1^ geometric mean; ^2^ MEP: monoethylphthalate; ^3^ MnBP: mono-*n*-butylphthalate; ^4^ MBzP: monobenzylphthalate; ^5^ DEHP: di(ethylhexyl)phthalate.
